# An update on recent progress of the epidemiology, etiology, diagnosis, and treatment of acute septic arthritis: a review

**DOI:** 10.3389/fcimb.2023.1193645

**Published:** 2023-05-02

**Authors:** Miao He, Djandan Tadum Arthur Vithran, Linyuan Pan, Haijin Zeng, Guang Yang, Bangbao Lu, Fangjie Zhang

**Affiliations:** ^1^ Department of Orthopaedics, Xiangya Hospital, Central South University, Changsha, Hunan, China; ^2^ National Clinical Research Center for Geriatric Disorders, Xiangya Hospital, Central South University, Changsha, Hunan, China; ^3^ Department of Emergency Medicine, Xiangya Hospital, Central South University, Changsha, Hunan, China

**Keywords:** septic arthritis, Pathogenic microorganism, antibiotics, arthroscopy, epidemiology

## Abstract

Acute septic arthritis is on the rise among all patients. Acute septic arthritis must be extensively assessed, identified, and treated to prevent fatal consequences. Antimicrobial therapy administered intravenously has long been considered the gold standard for treating acute osteoarticular infections. According to clinical research, parenteral antibiotics for a few days, followed by oral antibiotics, are safe and effective for treating infections without complications. This article focuses on bringing physicians up-to-date on the most recent findings and discussions about the epidemiology, etiology, diagnosis, and treatment of acute septic arthritis. In recent years, the emergence of antibiotic-resistant, particularly aggressive bacterial species has highlighted the need for more research to enhance treatment approaches and develop innovative diagnosis methods and drugs that might combat better in all patients. This article aims to furnish radiologists, orthopaedic surgeons, and other medical practitioners with contemporary insights on the subject matter and foster collaborative efforts to improve patient outcomes. This review represents the initial comprehensive update encompassing patients across all age groups.

## 1 Introduction

Acute Septic arthritis(ASA) is a rare and serious orthopedic emergency mainly affecting a single joint (5-10% of multiple joints) that, if left untreated, can lead to systemic sepsis and has a 16.3 percent death rate (Forlin and Milani, 2008; Ilharreborde, 2015; Montgomery and Epps, 2017; Tretiakov et al., 2019; Abram et al., 2020; Chan et al., 2020; Erkilinc et al., 2021; Momodu and Savaliya, 2022). The prevalence changes with age (Geirsson et al., 2008; Mathews et al., 2010; Kennedy et al., 2015; Maneiro et al., 2015), ethnicity (Morgan et al., 1996), and socioeconomic status (Gupta et al., 2001). In most circumstances, males are more likely to be affected than females (Al Saadi et al., 2009; Pääkkönen, 2017; Momodu and Savaliya, 2022).Although any joint is vulnerable to infection (Shirtlif and Mader, 2002), the knee is the most common site of infection (affecting almost half of all cases), followed by the hip, shoulder, elbow, and ankle. Rheumatoid arthritis, neonates, diabetes, heavy drinking, and old age are possible risk factors of ASA (Kaandorp et al., 1995; Mathews et al., 2010).

Hematogenous spread is the most common route for these infections to reach the joint space, while penetrating trauma or inoculation are potential triggers (Mathews et al., 2010; Ross, 2017).In addition to a patient’s medical history and physical examination, confirmation of a clinical diagnosis of septic arthritis needs the isolation of an infectious agent from synovial fluid. In cases when repeated joint aspiration has been unsuccessful, surgery is advised over medical treatment (Lane et al., 1990; Balabaud et al., 2007). Improper or delayed diagnosis and treatment may result in permanent joint damage and disability (Peters et al., 1992). *Staphylococcus aureus* is the most commonly cultured organism. It is followed by *Kingella kingae*, *Streptococcus pyogenes*, and *Streptococcus pneumonia*, depending on the patient age (Moumile et al., 2005). Antibiotic coverage should start in suspected cases when blood cultures and a serologic test and microscopic analysis of synovial fluid collected from the affected joint are the initial steps in diagnosing septic arthritis. White blood cell (WBC) count, C-reactive protein (CRP) level evaluation, erythrocyte sedimentation rate (ESR), and aerobic and anaerobic blood cultures comprise the serologic testing battery. Using arthrocentesis, it is also feasible to get a WBC count, neutrophil percentage, Gram stain, and culture from synovial fluid. In fifty percent of instances with septic arthritis, arthrocentesis yields positive culture results; nevertheless, this is insufficient to establish a diagnosis (Weston et al., 1991; Kocher et al., 2003; Quick et al., 2018).Standard treatment consists of irrigation and debridement of the affected joint, followed by intravenous antibiotics. This can be accomplished using either an open surgery technique (arthrotomy) or a minimally invasive minimally invasive technique (arthroscopy) (Perry, 1999). Arthroscopic management has supplanted open management as the treatment for septic arthritis (Butt et al., 2011).

However open management is still widely utilized and remains the preferred option for many hospitals. In order to limit the risk of lifelong disability, making a fast diagnosis and treatment plan for ASA patients is crucial. To provide the best possible care for these patients, doctors must have a comprehensive awareness of the patient’s medical history, the results of the physical exam, the diagnostic testing, and the available treatment options. These topics continue to be the subject of debate among experts, and Unfortunately, the literature has no consensus about the etiology, the best treatment, and the diagnosis method available for ASA patients. Therefore, in the current review study, we aim to provide an up-to-date on the recent epidemiology, etiology, diagnosis, and best treatment option for acute septic arthritis for physicians constantly facing these conditions in their daily work at the hospital.

## 2 Epidemiology

Many factors prevent us from having a complete picture of acute septic arthritis’s epidemiology. The rarity of the illness makes future research challenging due to their high overhead costs and other obstacles. Patients in whom septic arthritis is strongly suspected clinically may or may not have the diagnosis established microbiologically, historically leading to difficulties in disease categorization.

The annual incidence of ASA varies from 1 to 35 cases per 100,000 individuals in different countries (Gafur et al., 2008; Riise et al., 2008; Horowitz et al., 2011; Montgomery and Epps, 2017; Okubo et al., 2017; Welling et al., 2018; Safdieh et al., 2019; Cohen et al., 2020; Nossent et al., 2021; Momodu and Savaliya, 2022), with the United States having a rate of 4 to 10 cases per 100,000 individuals (Montgomery and Epps, 2017; Okubo et al., 2017; Okubo et al., 2017; Swarup et al., 2020; Erkilinc et al., 2021 ^).^ The incidence of the large joint is higher than small joints for septic arthritis and raised with age; the most commonly involved large joint was the knee and hand interphalangeal in the small joints (Mathews et al., 2010; Ilharreborde, 2015; Momodu and Savaliya, 2022). Staphylococcus aureus is the most common pathogen causing septic arthritis (Kennedy et al., 2015; Jung et al., 2018; McBride et al., 2020).

Children have a higher incidence of septic arthritis than adults (Donders et al., 2022). Individuals whose immune systems are compromised for whatever cause (sickle cell anemia, HIV/AIDS, chemotherapy patients). Individuals with diabetes mellitus, rheumatoid arthritis, recent joint surgery, a joint prosthesis, intra-articular injections in the past, a history of skin infections or cutaneous ulcers, HIV infection, or age over 80 are at increased risk (Margaretten et al., 2007; Horowitz et al., 2011).

Septic arthritis is on the rise, associated with an aging population, an increase in the number of invasive procedures performed, and an increase in the number of patients receiving immunosuppressive therapy. More research is needed on the topic to reach a consensus on the epidemiology of ASA.

## 3 Etiology

### 3.1 Pathogenic microorganisms

The prevalence and susceptibility of organisms that cause septic arthritis have not altered substantially during the past decades (Dubost et al., 2014). Staphylococcus aureus is the most prevalent organism for all age categories and risk groups, followed by Streptococcus (Kennedy et al., 2015).

The hip and knee are the joints most commonly affected by septic arthritis in children. As shown in **Table 1**, the most prevalent organism is *methicillin-sensitive Staphylococcus aureus* (MSSA), followed by methicillin-resistant Staphylococcus aureus (MRSA) and *Streptococcus pneumoniae* (Young et al., 2011). MSSA, MRSA, *group B streptococci*, *Klebsiella pneumoniae*, and gram-negative bacilli regularly infect infants younger than 3 months; *Neisseria gonorrhoeae* and *Candida* are rare pathogens in this age group (Ben-Zvi et al., 2019; Mooney and Murphy, 2019). Pathogens such as MSSA and MRSA, *group A streptococcus aureus*, and *Streptococcus pneumoniae* are common in infants and young children between 3 months and 5 years of age. *Haemophilus influenzae* type B and *Kingella kingae* are uncommon pathogens that commonly infect children aged 6 months to 4 years (Castellazzi et al., 2016; Villani et al., 2021). Pathogens such as *methicillin-resistant Staphylococcus aureus* (MRSA), *group A streptococcus, Streptococcus pneumoniae, Salmonella, Neisseria meningitidis, Neisseria gonorrhoeae, Pseudomonas aeruginosa, Candida, and anaerobic bacteria* other than group B are uncommon in children over 5 years old (Ben-Zvi et al., 2019). Children’s septic elbow is most commonly caused by Staphylococcus aureus (Bowakim et al., 2010).

**Table 1:** Pathogenic microorganisms for septic arthritis in all age groups.

AGES GROUP	COMMON PATHOGENS	RARE PATHOGENS
Infants younger than 3 months old	Staphylococcus aureus(MSSA and MRSA) group B streptococci Klebsiella pneumoniae gram-negative bacilli.	Neisseria gonorrhoeae Candida
Young children from 3 months to 5 years old	Staphylococcus aureus(MSSA and MRSA) group A streptococcus Aureus Streptococcus pneumoniae	Haemophilus influenzae type B
Children older than 5 years	Staphylococcus aureus(MSSA and MRSA) group A streptococcus.	Streptococcus pneumoniae group A And Beta hemolytic streptococcus Salmonella Neisseria meningitidis Neisseria gonorrhoeae Pseudomonas aeruginosa Candida anaerobic bacteria other than group B
Adults	Staphylococcus aureus, coagulase-negative Staphylococcus, Streptococcus, and Pseudomonas, and other Gram-negative bacteria.	

Pathogenic bacteria in adults may be directly tied to the patient’s medical history, physical state, drug misuse, etc., but the most prevalent pathogens are still *Staphylococcus aureus, coagulase-negative Staphylococcus, Streptococcus, Pseudomonas, and other Gram-negative bacteria*. In 233 cases of septic arthritis over 10 years, MSSA was the primary causal infection, but MRSA arthritis was rarely diagnosed (Clerc et al., 2011). In the United States, MRSA has become the leading cause of septic arthritis (Ross, 2017). Infections caused by MRSA are common in the elderly, intravenous drug users, and after orthopedic surgery. *Streptococcus pyogenes* is typically connected with autoimmune diseases, persistent skin infections, and trauma, but group B streptococci are frequently seen in the elderly, especially with diabetes, cirrhosis, and neurological disorders. Gram-negative bacilli infections account for 10–20% of cases of septic arthritis, and they frequently affect patients with urinary tract and intestinal infections and those with long-term implants. In the United States, injection drug use has become the most prevalent risk factor for septic arthritis. Septic arthritis in injected drug users is more frequently caused by MRSA, MSSA, *Serratia sp, Escherichia coli, Proteus, Klebsiella*, and *Enterobacter* and is more likely to affect the sacroiliac, acromioclavicular, sternoclavicular, and facet joints (Ross et al., 2020). Infrequently, anaerobic microorganisms are seen in diabetic patients, those who have received joint prosthesis implantation, and those who have sustained penetrating trauma. Women who develop septic arthritis during menstruation or pregnancy or who are sexually active should be evaluated for *Neisseria gonorrhoeae* infection (Clerc et al., 2011). Infection of a joint by MRSA appears to be related to worse outcomes (Ross, 2017). *Beta-hemolytic streptococci* predominantly caused streptococcal septic arthritis in older, multimorbid patients (Lotz et al., 2019).

The primary pathogens of septic arthritis produced by animal bites are the oral flora of injured animals and the flora of human skin, which includes various pathogens. *Pasteurella, Staphylococcus, Streptococcus*, and anaerobic bacteria are typical pathogens. Other uncommon pathogens include *Capnocytophaga*, which is transmitted by dog bites. Cat bites can potentially spread *Bartonella hansenii*. Pathogens of human bites are often aerobic gram-positive *cocci* (such as group A *streptococcus*) and *anaerobic bacteria*; *Pasteurella multocida* and *Eikenella eros* are rare pathogens (Moro-Lago et al., 2017; Gjika et al., 2019). *Pantoea agglomerans, Nocardia stellate, Sporothrix schenckii*, and purulent joints induced by consuming unpasteurized dairy products are the most prevalent pathogens of septic arthritis caused by horticultural workers or plant puncture wounds. Brucella is the most prevalent cause of inflammation (Smith et al., 2006; Clerc et al., 2011).

### 3.2 Pathological processes

The primary routes of joint infection include: 1) Hematogenous spread: pathogenic bacteria of the infection *foci* in other parts of the body spread to the joint through the blood circulation; hematogenous spread of infection is the most common etiology of shoulder sepsi^s^ (Sweet et al., 2019; Gramlich et al., 2020); 2) Adjacent infection: the pathogenic bacteria come from the skin and soft tissue infection around the joint or secondary intra-articular infection after osteomyelitis;3) iatrogenic: such as secondary infection following joint cavity puncture or medicament injection, or secondary intra-articular infection following joint replacement and per-articular fracture internal fixation and implantation, 4) traumatic, including trauma, stab wounds, and animal bites, resulting in joint soft tissue or joint infection after articular sac injury (Gjika et al., 2019; Couderc et al., 2020).

There are three stages to the pathophysiology of septic arthritis: 1) serous exudation stage: after pathogenic bacteria enter the joints, synovial congestion and edema, leukocyte infiltration, and serous exudation, there is typically no obvious damage to articular cartilage at this stage; if active treatment is administered, the exudate can be completely absorbed, the articular cartilage will not be destroyed, and the joint function will not be compromised. 2) Serous fibrinous exudation stage: the disease progresses, the exudate becomes turbid, the number of white blood cells and pus cells increases dramatically, synovitis worsens, vascular permeability increases and fibrin deposition causes articular cartilage rupture, ulcers, and shedding. The cartilage is uneven, resulting in poor joint function. 3) Purulent exudation stage: Inflammation progresses, the articular cartilage is involved, the subchondral bone and synovium are also destroyed, the soft tissues surrounding the joints can also be involved in cellulitis, the exudate is purulent, and the process is irreversible and causes severe dysfunction (Mathews et al., 2010; Couderc et al., 2020).

## 4 Clinical symptoms

Clinical signs might be modest or severe, especially in newborns and children are commonly atypical. The main symptoms include abrupt onsets such as chill, high fever, delirium, coma, and convulsions. It is more prevalent in youngsters, and some severe instances might be expressed as sepsis or septic shock (Synger, 2016). The joint’s local manifestations include heat, discomfort, dysfunction, mobility, and postural restriction. Very superficial joints, such as the knee and elbow, may exhibit noticeable redness and edema (Gottlieb et al., 2019). The patellar floating test may be positive. Deep joints, such as the hip and sacroiliac joint, may not exhibit visible swelling and fever, but joints may still be reluctant to move owing to pain, and a physical examination of diseased joints may be refused (Long et al., 2019; Couderc et al., 2020).

## 5 Laboratory test

Blood test: leucocytes are markedly elevated, generally greater than 10x109/L; leucocyte esterase for suspected septic arthritis in native joints has a high negative predictive value; for infection in a native knee, there should be few false-positive results (McNabb et al., 2017). Adopting leucocyte esterase may promote the quicker discharge of patients with negative results (Knapper et al., 2021). While erythrocyte sedimentation rate (ESR) and C-reactive protein (CRP) are also significantly elevated, elevated procalcitonin (PCT) is of greater diagnostic significance.

For knee, elbow, shoulder, hip, etc., if suspected of septic arthritis, an experienced physician should perform joint puncture and synovial fluid aspiration. Gram stain and culture of synovial fluid should be tested; genetic testing of pathogenic microorganisms should also be performed to rapidly identify the possible pathogenic bacteria in the synovial fluid or blood by polymerase chain reaction (PCR) or next-generation sequencing (NGS). WBC≥50,000/ml in a synovial fluid provides diagnostic significance for septic arthritis; however, only 5% of individuals with septic arthritis had WBC <50,000/ml in their synovial fluid (Coutlakis et al., 2002). Gram stains are positive in 40% to 70% of septic arthritis patients (Ross, 2017), although, in a study by McBride et al., 543 samples of septic arthritis in adults, only 40% had a positive synovial fluid culture (McBride et al., 2020). In another conducted by Daynes et al.,55% of 183 adult patients with native septic arthritis had positive synovial fluid cultures, while 54% of 65 patients with native septic arthritis had positive blood cultures; 91% of the blood culture results were identical to the joint fluid culture results. Other pathogens isolated from joint fluid cultures were MRSA, *Streptococcus* species, *Pseudomonas*, and others (Daynes et al., 2016). Automatic mPCR demonstrated at least equivalent performance to synovial fluid culture in diagnosing septic arthritis, with the tremendous advantage of a shorter time (Sigmund et al., 2019). Another study found that the PCR test for septic arthritis identified the bacterial etiology better (Coulin et al., 2021). In a single-center, cross-sectional investigation involving 95 patients, 16s rDNA PCR in synovial fluid did not improve the diagnostic performance of septic arthritis in native adult joints, particularly for Gram-positive cocci infections (Coiffier et al., 2019).

**Figure 1:**
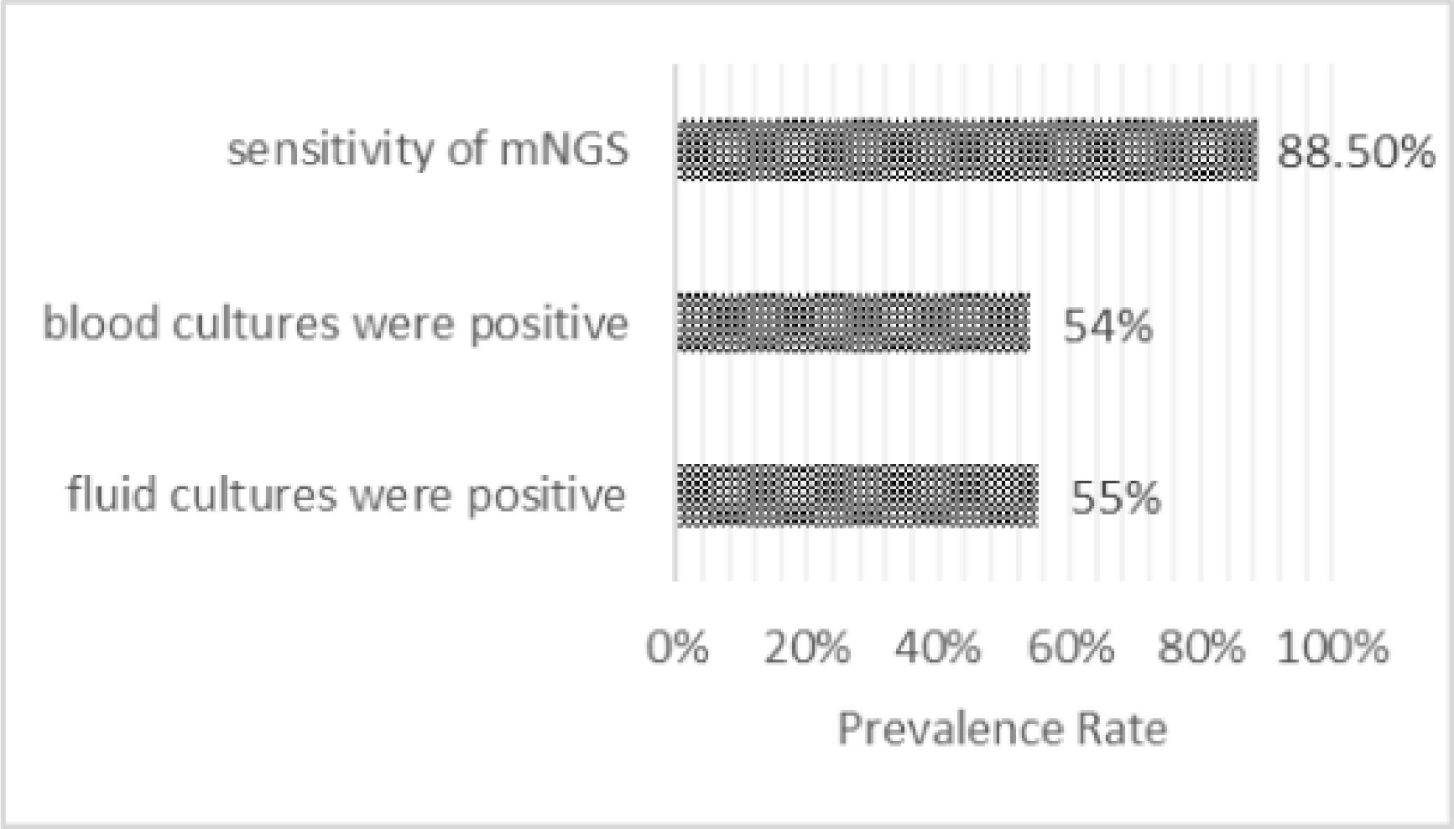
Compares the diagnostic value among mNGS, blood culture, and synovial fluid culture. (A total of 130 samples from 92 patients with osteoarticular infection account for the sensitivity of mNGS (Welling et al., 2018), while others were derived from another study altogether, 183 adult patients with septic arthritis (Kennedy et al., 2015)).

Because the pathogen culture has a high risk of false-negative results and is time-consuming, new timely diagnostic procedures are required. NGS has emerged as an enabling technological platform for detecting and taxonomic characterizing microorganisms in clinical samples from patients (Gu et al., 2019). Considering that almost all infectious agents contain DNA or RNA genomes, NGS has become an indispensable tool for detecting and classifying microorganisms in patient samples. NGS plays a crucial role in etiological discovery; the 2019-nCoV was initially discovered using NGS (Zhu et al., 2020). In patients with prosthetic joint infection, targeted antibiotic treatment for culture-negative infection based on metagenomic NGS (mNGS) results resulted in a favorable outcome. The mNGS test is reliable for identifying pathogens associated with culture-negative prosthetic joint infection (Tarabichi et al., 2018; Wang et al., 2020). Preoperative aspirated synovial fluid detected by mNGS provides more aetiological information than preoperative culture (Fang et al., 2021), and NGS is more accurate and sensitive than bacterial culture and serological indicators such as CRP, IL-6, and PCT for identifying prosthetic joint infection (Yin et al., 2021). In a study by Huang et al., a total of 130 samples from 92 patients with osteoarticular infections, the overall sensitivity of mNGS was 88.5%; however, the sensitivity of joint fluid samples was much greater, as shown in **Figure 1**. mNGS identified Coagulase-negative *Staphylococci*, Gram-negative Bacillus, Streptococci, Anaerobe, non-tuberculous *mycobacterium*, and *Mycoplasma* as pathogens. However, the sensitivity of mNGS was greater in antibiotic-treated samples than in microbiological cultures (Huang et al., 2020). NGS identified bacteria at a higher incidence in the skin, and deep tissue samples than conventional culture did in indigenous, non-infected subjects undergoing initial operations. Before NGS may be utilized reliably in orthopedic cases (Rao et al., 2020), it is necessary to evaluate which NGS data are clinically significant and which are false positives.

## 6 Imaging

Within one week of onset, X-ray and CT images of the articular structures are largely normal or reveal primarily soft tissue swelling, muscle space blurring, and joint space enlargement due to joint effusion. There is no obvious specificity, and with the improvement of economic living standards, there has been an increase in the number of patients seeking medical attention at the earliest stages of the disease, so X-rays and CT are limited in their ability to diagnose early septic arthritis. However, X-rays and CT are helpful in the differential diagnosis of acute osteomyelitis.

In patients with septic arthritis, MRI can detect the destruction of articular cartilage, characterized by rough, fuzzy, and shedding articular cartilage, and increased T2WI and PDWI signals at the damaged site, bone marrow edema. This is manifested as focal T2WI and PDWI signal increase of articular bones, and soft tissue edema is manifested as diffuse swelling of the soft tissue around the synovium, with increased T2WI and PDWI signals (Kang et al., 2020). In other cases, multiple abscess cavities can be seen in the surrounding soft tissue, which manifests as multiple cystic structures in the soft tissue around the joint. The enhanced scan shows a ring-enhanced lesion, purulent joints inflammatory synovial tissues that are hyperplasia, showing that the joint capsule synovium is thickened, the enhanced scan is enhanced, and even the intra-articular ligaments are wrapped. The joint cavity effusion manifests as the cavity’s long T1WI and long T2WI signals. Pediatric septic arthritis can be accompanied by metaphyseal osteomyelitis, epiphyseal osteomyelitis, or abscess, manifested by a low signal on T1WI and a high signal on T2WI. A lamellar periosteal reaction can sometimes be seen at the metaphysis (Karchevsky et al., 2004). Synovial enhancement, peri synovial edema, and joint effusion are most commonly correlated with the clinical diagnosis of a septic joint (Karchevsky et al., 2004). Independent associations of risk for septic arthritis include synovial fluid WBC ≥ 30,000/ml, bacteria reported on synovial fluid gram stain, duration of pain >2 days, and history of septic arthritis at any joint (Holzmeister et al., 2021). MRI should be utilized to diagnose suspected septic arthritis (Monsalve et al., 2015). As demonstrated in **Table 1**, the differential diagnosis included joint tuberculosis, rheumatoid arthritis, rheumatoid arthritis, gouty arthritis, and osteoarthritis synovitis.

## 7 Antibiotics therapy

Early and appropriate administration of antibiotics (without waiting for bacteriological results) and immobilization of the afflicted limb are required. Antibiotics require thoroughly evaluating the patient’s medical history and clinical symptoms. If there is sepsis or septic shock, antibiotics must be de-escalated empirically. *Staphylococcus aureus* has the highest resistance to penicillin, reaching 96%, and is sensitive to vancomycin, teicoplanin, linezolid, rifampicin, amikacin, Gentamicin, and ciprofloxacin; *Klebsiella pneumoniae* and *Escherichia coli* have a higher sensitivity to meropenem and imipenem, cephalosporin third generation, ciprofloxacin, and tetracycline. Nevertheless, rifampicin, amikacin, Gentamicin, tetracycline, and ciprofloxacin have relatively large side effects, and children are generally unsuitable for use (Clerc et al., 2011).

No substantial rise in resistance microorganisms causing septic arthritis was identified in a retrospective research period of 15 years involving 85 patients with septic arthritis; these results lend support to the use of narrow-spectrum antimicrobials on an empirical basis for septic arthritis (Ben-Chetrit et al., 2020).

For babies younger than three months, empiric therapy for septic arthritis should target *Staphylococcus aureus, Streptococcus, and Gram-negative bacilli*. The preferred treatment is nafcillin or vancomycin coupled with cefotaxime or ceftazidime (if *Pseudomonas* is suspected). *Staphylococcus aureus* and other gram-positive organisms should be the focus of empirical therapy for septic arthritis in children 3 months (for example, *Group A Streptococcus, Streptococcus pneumoniae*). Clindamycin, or a cephalosporin of the first generation, is a suitable treatment. If the youngster is not immunized against *Haemophilus influenzae*, ampicillin or amoxicillin should be administered. If just 10%-15% of the isolated bacteria are MRSA and the child is hemodynamically stable, cefazolin and nafcillin can be explored for treatment. If 10%-15% of the isolated bacteria are MRSA, it is advised to administer clindamycin and vancomycin. Consider *Streptococcus pneumoniae* resistant to penicillin, vancomycin, and clindamycin. If the infection is highly suspected to be Gram-negative *bacilli*, providing cephalosporins of the second or third generation is advisable. Cephalosporins (such as cefazolin, cefotaxime, and ceftriaxone) can typically be used to treat Chinchilla infections, although they are resistant to vancomycin and typically resistant to clindamycin and *staphylococcus* Resistant to a penicillin (nafcillin) (Erkilinc et al., 2021). Corticosteroids may enhance the proportion of patients without discomfort and the proportion of patients with the normal function of the afflicted joint at 12 months, as well as decrease the number of days children require antibiotic treatment (Delgado-Noguera et al., 2018). Therefore, preoperative antibiotics should be avoided in children after septic arthritis was not proven to be diagnosed. Therefore, antibiotics should not be administered to children with an unconfirmed diagnosis of septic arthritis. Their prescription delays the diagnosis and ultimate surgery, causing extra problems and washouts (MacLean et al., 2015).

For empirical coverage of large-joint septic arthritis, Amoxicillin/clavulanate or cefuroxime would be sufficient for large-joint infections. Infections of tiny joints in diabetics would be much better treated with a broad-spectrum antibiotic. Systematic coverage of MRSA is not justified, although known carriers should be considered (Clerc et al., 2011).

**Figure 2:**
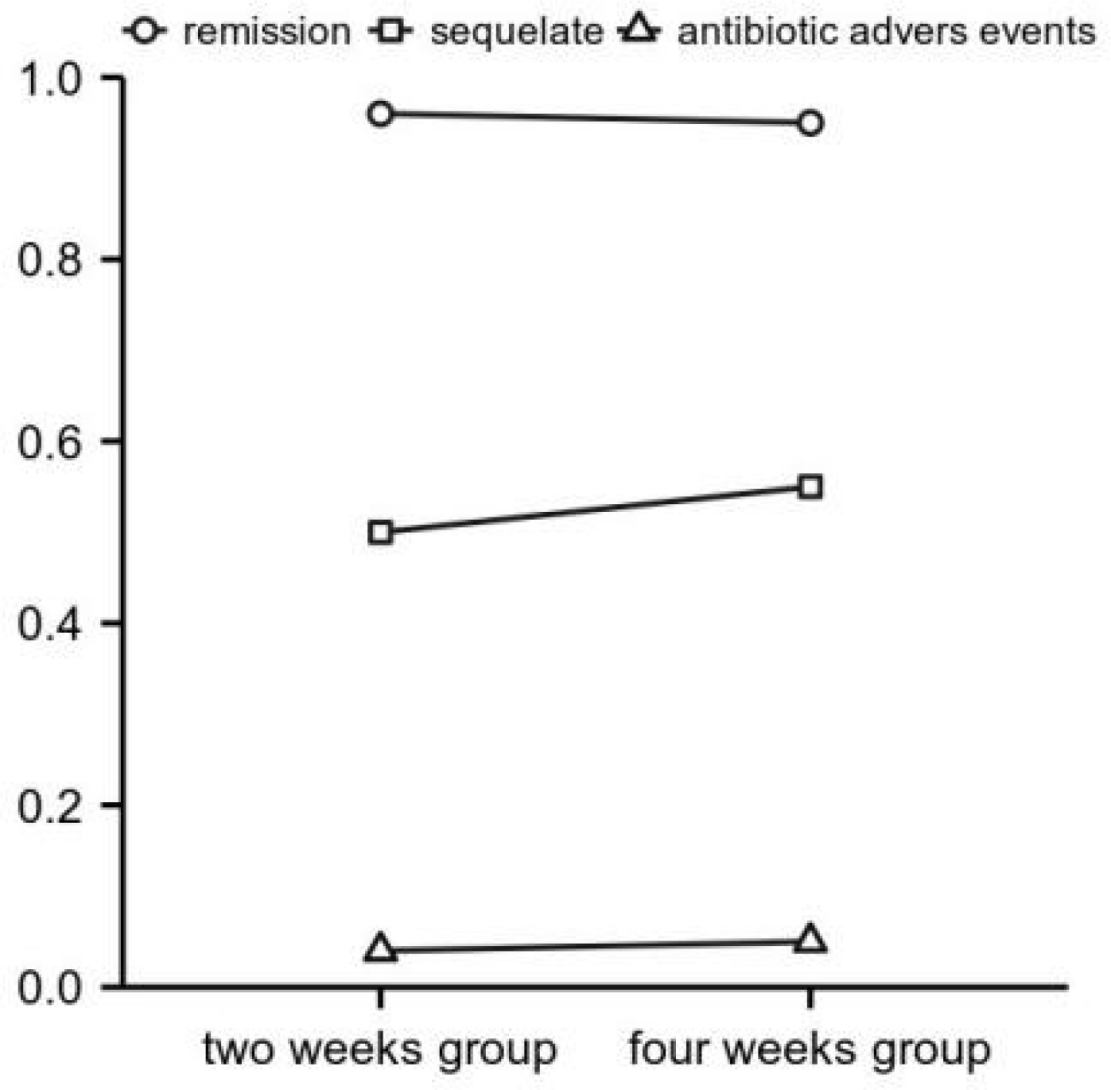
Outcomes of arthritis therapy of the hands and wrists (subgroup analysis). X-axis: study outcome parameters. Y-axis: number of corresponding episodes with proportions and absolute number of cases (Ross, 2017).

Antibiotic courses of 3 to 4 weeks are usually adequate for uncomplicated bacterial arthritis. Treatment duration should be extended to 6 weeks if there is imaging evidence of accompanying osteomyelitis (Ross, 2017). In a prospective, unblinded, randomized, non-inferiority study comparing either 2 or 4 weeks of antibiotic therapy after surgical drainage of native joint bacterial arthritis in adults, 2 weeks of targeted antibiotic therapy is not inferior to 4 weeks regarding cure rate, adverse events, or sequelae. It leads to a significantly shorter hospital stay, as **Figure 2** showed, at least for hand and wrist arthritis (Gjika et al., 2019). For uncomplicated native joints with septic arthritis of the hand, current evidence suggests that a 2-week course of antibiotic therapy following surgery cured septic arthritis (Sendi et al., 2020).

## 8 Surgical procedures

Once a patient is diagnosed with septic arthritis characterized by joint discomfort, reduced mobility, and inability to bear weight, he or she should have surgery or arthroscopy for irrigation and debridement to drain purulent fluid (Cargnelli, 2015; Memon et al., 2018). In the absence of clinical sepsis, early joint drainage does not appear to improve the risk of sequelae for native septic arthritis compared to delayed drainage (Lauper et al., 2018). Nevertheless, pathogen type and comorbid diseases did not affect the length of stay (Daynes et al., 2016). In a retrospective study of 79 patients with septic knee arthritis, effective treatment needed an average of 1.3 operations. With arthroscopic irrigation and debridement, most patients with septic knee arthritis need only one surgical surgery to eliminate the infection. From the symptom beginning to surgery, the necessity for several interventions increases (Dave et al., 2016).

Arthroscopic surgery can treat joint cavity infections with debridement under the microscope, continuous closed lavage and drainage, harmful bacteria disappear rapidly, infection control is dependable, antibiotics are employed for a short period, and the efficacy is satisfactory. Arthroscopic treatment for acute native knee septic arthritis was a more successful index surgery, required fewer total irrigation procedures, and had a lower reinfection rate and low initial inflammatory response than existing treatment (Peres et al., 2016). After arthroscopic treatment, the patient’s long-term range of motion was much greater (Johns et al., 2017). A Systematic Review concluded that arthroscopic native hip irrigation and debridement for septic arthritis appear to comprise a safe and effective treatment option for selected patients (Cargnelli, 2015). Arthroscopic management may be a safe option for treating hip septic arthritis with potentially limited morbidity (Lee et al., 2014; Khazi et al., 2020). For septic shoulder arthritis, most patients with septic arthritis of native shoulders were effectively treated with a single arthroscopic irrigation and debridement (Joo et al., 2020). However, systematic reviews showed that arthroscopic surgery and open Arthrotomy have similar efficacy, although arthroscopic native shoulder septic arthritis had the results of pain alleviation and joint movement recovery; unfortunately, there was a high reoperation rate (Memon et al., 2018; Bovonratwet et al., 2019).

Continuous irrigation of the joint cavity is an option for patients undergoing unconditional arthroscopic surgery or those with shallow joints. It is necessary to make two holes in the joints, one for the intake tube and one for the outlet tube. Daily perfusion of 2000-3000 ml antibiotic-containing saline through the catheter, the outlet tube has a clear liquid, and the lavage can be stopped after culture without bacterial growth, but drainage The tube still needs to be sucked for several days until the drainage volume is reduced to no fluid outflow, and the local symptoms and signs have disappeared (Johns et al., 2017). Surgical joint incision and drainage are required for intra-articular infection caused by the prosthesis around the joint (Smith et al., 2006).

## 9 Treatment outcomes and prognosis

A retrospective cohort analysis included 12132 patients with septic arthritis who underwent arthroscopic knee washing in England between 1997 and 2017. Among 10 195 (84%) patients with septic arthritis as the primary admission diagnosis, the 90-day mortality rate was 7.05 percent, but 22.69% in 1842 patients older than 79. The 1-year rates for arthrodesis, amputation and arthroplasty were 0.13%, 0.40%, and 1.33%, respectively. Within 15 years of follow-up, 8.76% of patients had undergone arthroplasty, equating to a risk of arthroplasty six times that of the general population (Abram et al., 2020).

In contrast to the knee, it is envisaged that septic arthritis of the shoulder will result in a severe loss of function (Gramlich et al., 2020). Long-term recurrence of glenohumeral fractures following clinically effective therapy Joint septic arthritis is uncommon, and few patients get elective arthroplasty after septic shoulder arthritis (Sweet et al., 2019). If the therapies were ineffective, the death rate at 30 days was 2% and increased to 6% after 90 days (Kennedy et al., 2015). Treatment failure was independently linked with joint size, age, intra-articular non-arthroplasty prosthesis, and surgical procedures performed. Small-joint septic arthritis has a better prognosis than large-joint septic arthritis and may be treated safely with shorter antibiotic courses (McBride et al., 2020). Injection drug use is a growing cause of septic knee admissions and is related to greater rates of death, recurrent arthroscopic or open irrigation, and debridement (Oh et al., 2018). A retrospective single-center study of 186 patients with native septic arthritis revealed that Staphylococcus aureus infection, endocarditis, and the involvement of joints difficult to access with needle drainage predict treatment failure and that age, baseline leukocyte count, bacteremia, diabetes, and chronic renal failure predict mortality (Maneiro et al., 2015). In Old age, anginosus group streptococci, *enterococci*, and polymicrobial infections predicted poor outcomes, while antibiotic treatment duration can likely be shortened (Kaandorp et al., 1995). MRSA was identified as a risk factor for an unplanned return to the operating room after arthroscopic treatment (Jaffe et al., 2017). *Staphylococcus aureus* is an independent risk factor for the recurrence of infections after surgical treatment of shoulder septic arthritis (Böhler et al., 2017).

Patients with septic arthritis of the shoulder frequently experience substantial systemic complications regardless of the treatment method. Septicemia was a common complication among all treatment groups, with cultures most frequently indicating *Staphylococcus aureus* as the causative organism (Jiang et al., 2017). Adults with a history of inflammatory arthropathy, involvement of a large joint, a synovial-fluid nucleated cell count of >85.0 × 109 cells/L, infection with S. aureus, or a history of diabetes had a higher risk of failure of a single surgical debridement for acute septic arthritis and requiring additional surgical debridement (Hunter et al., 2015).

## 10 Conclusions

Notwithstanding the pressing need for prompt diagnosis and intervention, a dearth of comprehensive data pertaining to various facets of its management at an advanced level exists. Septic arthritis of an acute nature primarily impacts the joints of the hip and knee. Staphylococcus aureus and Streptococcus are the predominant pathogenic microorganisms. Diagnosis of septic arthritis is typically straightforward based on clinical manifestations, laboratory findings, and MRI. Early and appropriate administration of antibiotics for 2-4 weeks is imperative. Arthroscopic surgery is a minimally invasive approach that yields favorable outcomes for treating septic arthritis. In cases where the ordering providers encounter challenging scenarios, effective communication between the radiologist and the providers can be facilitated by the radiologist’s up-to-date knowledge of the latest research. This can enable the radiologist to provide valuable insights and recommend a joint aspiration procedure. A cooperative effort among radiologists, orthopaedic surgeons, and other medical professionals is necessary to enhance patient outcomes.

## Author contributions

MH, DTAV, and PL have contributed equally to this work and shared the first authorship did the writing—original draft preparation, Conceptualization, Writing ‐ review & editing and Data curation and Software; HZ and GY did Methodology, Data curation and Investigation; BL and FZ did the Supervision, the Project administration and Validation. All authors contributed to the article and approved the submitted version.
